# Role of integrative structural biology in understanding transcriptional initiation

**DOI:** 10.1016/j.ymeth.2019.03.009

**Published:** 2019-04-15

**Authors:** Michael J. Trnka, Riccardo Pellarin, Philip J. Robinson

**Affiliations:** a Department of Pharmaceutical Chemistry, University of California San Francisco, San Francisco, CA, USA; b Institut Pasteur, Structural Bioinformatics Unit, Department of Structural Biology and Chemistry, CNRS UMR 3528, C3BI USR 3756 CNRS & IP, Paris, France; c Department of Biological Sciences, Birkbeck University of London, Institute of Structural and Molecular Biology, London, United Kingdom

**Keywords:** Integrative structural determination, RNA polymerase II, Transcriptional initiation machinery, Cross-linking mass spectrometry, Multi-subunit complex architecture, Computational molecular modelling, Cryo-EM

## Abstract

Integrative structural biology combines data from multiple experimental techniques to generate complete structural models for the biological system of interest. Most commonly cross-linking data sets are employed alongside electron microscopy maps, crystallographic structures, and other data by computational methods that integrate all known information and produce structural models at a level of resolution that is appropriate to the input data. The precision of these modelled solutions is limited by the sparseness of cross-links observed, the length of the cross-linking reagent, the ambiguity arisen from the presence of multiple copies of the same protein, and structural and compositional heterogeneity. In recent years integrative structural biology approaches have been successfully applied to a range of RNA polymerase II complexes. Here we will provide a general background to integrative structural biology, a description of how it should be practically implemented and how it has furthered our understanding of the biology of large transcriptional assemblies. Finally, in the context of recent breakthroughs in microscope and direct electron detector technology, where increasingly EM is capable of resolving structural features directly without the aid of other structural techniques, we will discuss the future role of integrative structural techniques.

## Highlights

• Integrative methods spearhead structural studies of Mediator complex.
• 3D architectural models combining data from diverse techniques.
• Exhaustive computational sampling produces best macromolecular models.
• Complex modularity and transferability encoded in modelling scoring function.
• Structure studies of dynamic and disordered transcription complexes tractable.

## 1 Introduction

The goal of structural biology is to derive detailed functional and mechanistic information on a biomolecule from the arrangement of its constituent atoms. Since their introduction in the 20th century the two techniques at the forefront of structural biology, X-ray crystallography and electron microscopy (EM), have undergone seismic changes leading to an explosion in the numbers of scientists practicing structural biology and the pursuit of biological targets of ever-increasing complexity. X-ray crystallography has trended from focusing on small soluble proteins and individual protein domains [1], [2], [3] to large multi-subunit complexes and challenging membrane-associated targets [4], [5], [6], [7]. In contrast electron microscopy has seen an evolution from massive, highly symmetrical macromolecules [8], [9], to ever-smaller and asymmetric targets [10]. This evolution reflects an important underlying principle of biology; namely, that biological processes often occur in the context of large and asymmetrical protein, lipid-associated or nucleoprotein assemblies. The function of these assemblies is often dependent on a large set of more transient biomolecule interactions and on significant levels of domain motion, which act as sources of compositional and conformational heterogeneity, respectively. Such biological heterogeneity has provided significant technological challenges and has frequently exposed the limitations of individual structural biology techniques. For example, the formation of a highly populated and ordered macromolecular crystal lattice, which forms the basis for high-resolution X-ray diffraction data, is dependent on the deposition of a population of macromolecules that are identical in respect to both their composition and conformation. Sources of heterogeneity lead to lattice ‘poisoning’ that attenuate the intensity of useful Bragg diffraction. Likewise, in the context of electron micrographs, which capture images of individual macromolecules with low signal-to-noise, sources of macromolecular heterogeneity lead to increased errors in the assignment of projection angle [11], [12] and put extra demands on the computational routines designed to separate particles into distinct structural classes [13].

Due to this limitation of individual structural techniques, studies of large macromolecular assemblies have often been limited to distinct subassemblies, which suffer less from problems due to heterogeneity and therefore can be solved at higher resolution. Integrative structural biology approaches were developed to overcome the shortfalls of individual structural techniques and provide a framework for combining the data from multiple structural approaches to form a more complete picture of dynamic biological assemblies. A branch of this integrative approach, Integrative Structural Determination (ISD), attempts to use as much of the relevant biochemical and biophysical data about a macromolecular complex as possible to generate three-dimensional structures (models). ISD merges data sets that individually cannot lead to the unambiguous (e.g. atomistic) structural determination of the whole macromolecular complex. ISD exploits the mutual synergy and consistency of the datasets in such a way that the resulting model precision is higher (and therefore more informative) than the precision of the models generated by each individual dataset. In the ISD approach, datasets are encoded into a scoring function which is able to rank models according to how compatible they are to the input data. The configurational space of the represented complex is then extensively and thoroughly sampled. Best-scoring models (the ensemble of solutions) are validated and analyzed to assess their quality and build testable hypotheses [14], [15].

Importantly, two main problems can limit an effective ISD approach: the lack of data and the incomplete sampling of the models. The former can be due to the instability of the experimental sample or technological limitations, while the latter is related to the huge number of degrees of freedom of large protein complexes. To overcome these issues, the ISD approach can exploit two important principles that are intrinsic to the hierarchical architecture of assemblies: the *transferability* of datasets and the *modularity* of the structure (Fig. 4). The transferability assumes that the bulk of the data is robust, irrespective of the functional state of the complex. Using this principle, one can collect data on a functional state where the protein complex is more stable, and use the data to model the functional state of interest. The modularity principle assumes that the complex is formed of several stable and independent sub-complexes, whose architecture varies only modestly upon changes in the functional state. The modularity allows investigators to assign the data to distinct components of the system in a divide-and-conquer fashion, allowing a more efficient model sampling.

Over the last decade the ISD approach has been remarkably successful in modeling the architectures of large, fundamentally important complexes whose structures seemed intractable to single techniques such as those involved in transcription (pre-initiation complex, Mediator, TFIIH, etc.), translation (ribosome, elF3) and transit across the nucleus (nuclear pore complex) [16], [17], [18], [19], [20], [21], [22], [23], [24]. Many of these applications assumed the modularity and transferability principles.

In this review we will provide a general description of the integrative modeling pipeline and then review how it has been implemented to provide novel details of a number of complexes involved in RNA polymerase II (RNAPII) transcription. Finally, we will provide a discussion of the future of integrative modeling in the context of recent transformational developments in cryo-EM.

## 2 RNAPII transcription initiation machinery

The initiation of transcription from eukaryotic genes depends on the assembly at gene promoters of a massive pre-initiation complex (PIC) involving RNAPII and the general transcription factors (GTFs) TFIIA, -B, -D, -E, -F, -H and -S. This 31-protein assembly includes proteins that recognise and bind conserved regulatory promoter DNA elements upstream of the transcriptional start site, distort the DNA to direct its path along the active site cleft of RNAPII and apply DNA helical torsion in an ATP-dependent manner to melt the DNA duplex and promote single-stranded template DNA binding within the RNAPII active site. The Megadalton Mediator complex, traditionally classified as a transcriptional coactivator complex, also plays an essential role in the initiation pathway [25], [26]. Mediator binds transcriptional activator proteins and supports the stimulation of transcription at inducible gene promoters in response to activator UAS binding [27], [28]. The augmented transcriptional response to activators depends on the integrity of the unstructured C-terminal domain (CTD) of RNAPII [29], [30], [31], which is highly conserved across eukaryotes and composed of tandem heptapeptide repeats. Mediator binds to the CTD and in doing so acts as a bridge between cellular regulatory signals and the basal transcriptional machinery. Mediator-RNAPII interactions are responsible for increasing RNAPII levels and transcriptional output at gene promoters in vivo [32], [33]. Mediator binds to unmodified CTD repeats [34] and RNAPII molecules with unmodified CTD sequence are exclusively recruited to the PIC [35]. Upon full PIC assembly the CTD is brought together with the CTD kinase TFIIK (a submodule of TFIIH), and the resulting CTD phosphorylation correlates with loss of the Mediator-RNAPII interaction and the transition to the elongation phase of the transcription cycle (RNAPII promoter escape) [36], [37]. A striking feature of the gene activation pathway is the modular organisation of the transcriptional apparatus involved. Most of the polypeptides constituting the PIC are members of multi-subunit sub-assemblies that must be brought together as ‘ingredients’ for efficient transcriptional initiation. This modularity provides ample opportunities to regulate this early stage of transcription, such as in the example described above, where post-translational modification of the CTD is used to regulate the stability and residency of factors at the promoter. A further layer of regulation may exist through the compartmentalization of the transcription apparatus into so-called membrane-less compartments within the nucleus. Recent data have shown that the Mediator complex plays a critical role in bridging enhancer and promoter elements in the genome [38], [39]. An emerging view in the field is that in Metazoans the colocalization of super-enhancer-bound transcription factors, coactivators such as Mediator, and the promoter-associated general transcription machinery may in part be driven through a process termed biomolecular condensation, where multivalent interactions involving intrinsically disordered domains contribute to a phase-separated state producing regions with a high local concentration of essential transcription components. Support for this idea comes from recent observations of nuclear puncta in which Mediator and RNAPII colocalise with super-enhancer elements in structures that have properties of phase-separated condensates such as sensitivity to a non-specific aliphatic alcohol, 1,6-hexanediol [40], [41], [42]. A comprehensive biochemical and structural dissection of the interaction between the yeast activator Gcn4 and its Mediator target, Med15, has described a large heterogenous “fuzzy” complex comprised of multiple low affinity hydrophobic interactions that interact additively in a non-specific free-for-all manner to increase the overall binding affinity [43], [44]. The fact that the activation domains of transcription factors such as Gcn4 are archetypical intrinsically disordered regions brings into question the contribution of the “fuzzy” hydrophobic interface to the formation of membrane-less transcriptional condensates in vivo [45].

## 3 Novel biological findings from integrative structural biology-based studies of transcription complexes

### 3.1 Introduction to integrative structural determination

**Fig. 1:**
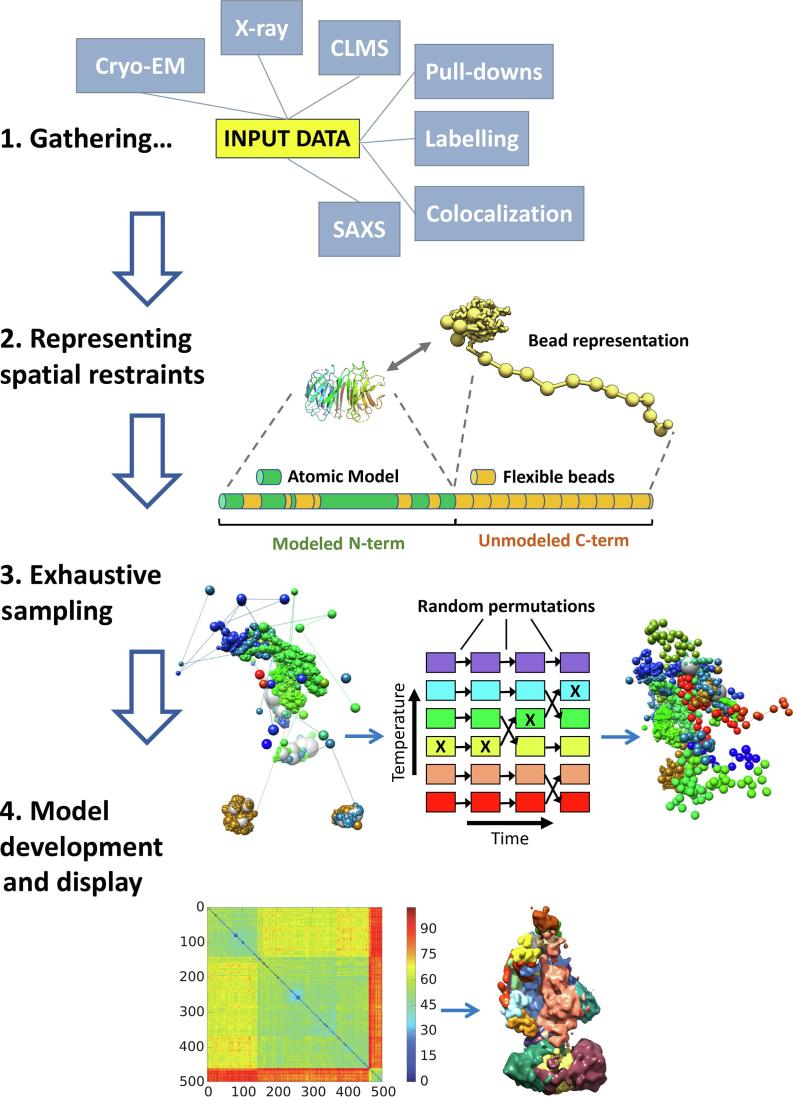
Representation of the multiple stages of integrative structural determination.

The ISD workflow follows a series of well-defined steps (Fig. 1) [15], [46]. First, all available primary data for the system of interest are gathered (see Section 4.4 for details). Datasets generally used by ISD include low-resolution three-dimensional EM reconstructions, small-angle X-ray scattering (SAXS), atomic-level data for subunits or domains, cross-linking mass spectrometry (CLMS), affinity pulldowns, labeling and co-localization experiments. The second stage involves generating a multi-scale representation of all components of the multi-subunit assembly and translating data into spatial restraints (see Section 4.5 for details). For certain domains in which atomistic structural information in the form of either homology models or crystal structures are available, these regions will be represented at the atomic or residue level as fixed rigid-body structures. Other domains will have no atomic resolution structural information and will be represented with chains of coarse-grained beads, with a suitable excluded volume, and connected by the continuity of the polypeptide chain. The third stage in the modeling workflow involves performing exhaustive computational sampling of the configurational space in order to identify a population of model solutions that best satisfy the full set of input restraints (see Section 4.6 for details). In practice this is achieved by computing parallel modeling trajectories or replicas (Replica Exchange) [47], each starting from a randomised starting configuration. Upon convergence of the computational sampling the ensemble of top-scoring solutions is classified by structural similarity to detect discrete structural subpopulations (see 4.7, 4.8 for details). At this stage subunit densities are calculated for each subpopulation cluster and information relating to how precisely each subunit in the model has been localised can be generated and graphically represented. Also, information relating to protein domain proximities in the complex can be extracted and graphically represented to reveal interesting biology and form the basis for future biochemical characterisation. To validate the resulting models a series of tests are performed that are designed to check restraint data robustness and the completeness of sampling (see Section 4.9 for details).

Over the last few years, we exploited the modular properties of the transcription machinery to model the architecture of increasingly complex transcription factor assemblies using ISD. We proceeded hierarchically, building on structural studies of isolated subcomplexes, and eventually characterized the large Mediator-bound PIC (Med-PIC).

### 3.2 Mediator Head module structure

**Fig. 2:**
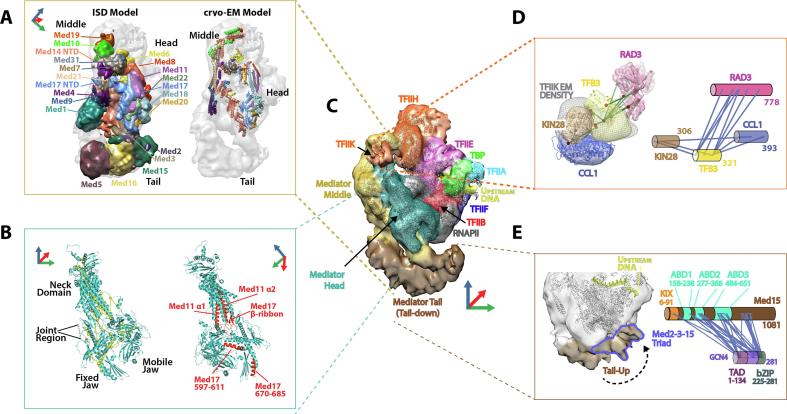
Novel RNAPII transcription complex biology determined using integrative structural methods. (A) Architecture of the yeast Mediator complex. Mediator subunit localization densities (left side) determined for the free Mediator using ISD methods, docked into Med-PIC cryo-EM map (grey transparent density). Atomic model of the yeast Head and Middle lacking Med1 (right side) from recent cryo-EM studies [77] oriented for direct comparison to the docked Mediator ISD model. Coloured coordinate axes show the orientation of the models relative to the Med-PIC cryo-EM map (panel C). (B) Integrative structural solution of the Mediator Head module. A cross-link dataset (left side; yellow colour) was used to guide model building into X-ray crystallographic map density at 4.2 Å resolution. Alternative view of the Head module (right side) showing regions in which revisions to sequence assignment or novel model build were supported by CLMS distance restraints (red colour). (C) cryo-EM structure of the complete Mediator-bound transcriptional pre-initiation complex [18]. Density for RNAPII, general transcription factors, promoter DNA and individual Mediator modules is coloured to facilitate map interpretation. Coloured coordinate axes provide a reference to orient structural components in other figure panels. (D) Integrative structural determination of TFIIK architecture. TFIIK subunits (Kin28, Tfb3 and Ccl1) were localized to novel Med-PIC cryo-EM map density (grey mesh) by integrating CLMS and EM restraints (left side). Modeling was supported by docking of a homology model for the TFIIH ATPase RAD3 into the cryo-EM density. Cross-links between TFIIK subunits and RAD3 together with EM restraints supported the integrative structural determination of TFIIK subunit localisation maps (solid densities). The position of homology models that contributed to the multi-scale representations of TFIIK subunits are shown positioned within their corresponding localization densities. Schematic representation of the inter-subunit cross-links that provided distance restraints for TFIIK integrative modeling (right side). (E) Dynamics and GCN4 cross-linking of the Mediator Tail module. Cryo-EM map from a particle sub-population in which the Mediator Tail module was in the ‘Tail-Up’ conformation (left side). In the ‘Tail-Up’ conformation the Tail moves closer to RNAPII and the upstream promoter DNA with bound activators. Schematic representation of the cross-links between the activator GCN4 and its main target within Mediator, Med15. The transcriptional activation domain and DNA-binding bZIP domain of GCN4 are indicated. The four Med15 domains known to be targets of GCN4 (KIX domain and activator binding domains 1–3) are indicated. (For interpretation of the references to colour in this figure legend, the reader is referred to the web version of this article.)

Biochemical and structural studies have established that the Mediator complex is composed of three modules, the Head, Middle and Tail modules [48], [49], that have a roughly equal share of the 21 subunits that comprise the core yeast Mediator complex. The subunit composition of each module was defined by a combination of biochemical and genetic analyses [49], [50], [51], [52], [53], [54]. Genetic screens for suppressors of a truncated RNAPII CTD identified a number of so-called SRB genes, five of which encoded core Mediator subunits [27], [50], [55]. Four of the five core Mediator SRB gene products were found to reside in the Head module, a 220 KDa assembly of seven proteins, most of which were found to be essential for yeast viability. Taken together with biochemical experiments showing that the full Mediator displays a robust CTD binding response [27], [50], [51], these genetic observations suggested a functional interaction between the CTD and the Head module with a direct interaction likely. Under non-permissive conditions, conditional mutations in the largest Head module subunit, Med17 (Srb4), lead to a dramatic global reduction of mRNA levels brought about through a destabilisation of the Mediator complex [25], [26], [56]. Therefore, the Mediator complex acts through the Head module to perform an essential role in the pathway to transcriptional activation at most eukaryotic gene promoters. A number of studies set out to elucidate the structural basis of Head module activities in transcription. The structure of a Med18-Med20 complex, encoded by two of the SRB genes, was the first atomic level information available, but provided little framework for understanding Head module function [57]. Structural information was extended to include the full seven-subunit Head module isolated from recombinant preparations of the yeast complex using X-ray crystallography [58]. From the resulting 4.2 Å density maps an architectural polyalanine model, composed mostly of alpha helices, revealed roughly 60% of the primary sequence (PDB: 3RJ1). The structure showed that the Head module can be divided into three independent structured domains, two termed the “Mobile Jaw” and “Fixed Jaw” based upon EM observations [59], which are separated from a third highly helical “Neck” domain by a mainly unstructured “Joint” region (Fig. 2B). In the absence of full chain connectivity and fully resolved side chain density, the very challenging job of assigning and threading subunit sequence into the secondary structure elements of this architectural model was guided by structure predictions and selenomethionine difference density peaks [58].

Shortly after publication of the recombinant yeast Head module we solved the structure of the native yeast Mediator Head module both alone and bound to the RNAPII CTD [34]. A strong set of phases from multiple isomorphous replacement with anomalous scattering (MIRAS) experiments generated maps that, whilst at a similar resolution to that achieved for the recombinant Head module, revealed features not seen previously such as domain connectivity in the central “Joint” region and large sections of β-sheet within both the “Neck” and the “Fixed Jaw” domains. Within these maps elongated CTD density comprising around four heptad repeats was found to follow a path across a highly conserved surface of the “Neck” domain. Modeling the new Head module density would provide a model that accounted for 80% of the primary sequence. However, the following challenges presented themselves during early model-building efforts: (1) sequence assignment for helices in subunits Med11 (“Neck”) and Med17 (“Fixed Jaw”) conflicted with the existing Head module structure (PDB: 3RJ1), (2) sequence ambiguities for inter-domain connectivity within the “Joint” region remained. To address these challenges, we employed an integrative approach where CLMS derived distance restraints were used to help revise sequence assignments and accurately model domain connectivity within the “Joint” region.

Revising the Med11 sequence assignment involved correctly determining the directionality of Med 11N-term helix 1 (α1) and helix 2 (α2), which are core elements in a 10-helix bundle within the “Neck” region. Cross-links within the “Neck” were consistent only with a Med11 assignment in which the directionality of Med11 α1 and α2 were reversed from that proposed in 3RJ1 (Fig. 2B). Such a reversal also positioned the C-terminal end of Med11 α2 within continuous map density that connected to the Med11 C-term, and was consistent with a high-resolution X-ray crystal structure of an isolated Med11-Med22 four-helix bundle [60].

Revising C-terminal Med17 sequence involved, amongst other things, determining the correct sequence connectivity between helices that were previously modelled as unconnected elements and whose sequence had been assigned based mostly on a sparse collection of selenomethionine difference peaks. CLMS analysis supported the reassignment of Med17 C-terminal 597-611 and 670-685 helices and surrounding sequence (Fig. 2B). For example, based upon the 3RJ1 Med17 sequence assignments, cross-links between Med17 K589, K601 and K608 with five surrounding lysines gave an average Cβ cross-link distance of 40.9 Å, significantly greater than the typical violation cut-off of 30 Å. After reassignment the average Cβ cross-link distance for the same collection of cross-links was 20.6 Å.

A cluster of SRB mutations map to residues within the “Joint” region of the Head module [50], [55]. A first step to understanding the structural basis of these mutations was to model this region of the structure, since it remained completely unmodeled in 3RJ1. Despite the presence of clear and continuous protein backbone densities, the combination of low secondary structure content, elevated B-factors and insufficient map resolution to position side-chain densities made “Joint” region modeling particularly challenging. Ambiguities were resolved by incorporating constraints from CLMS analysis into the molecular modeling workflow. A relatively large number of cross-links mapped to the “Joint” region, with a particular concentration in a Med17 hotspot comprised of six independent Med17 cross-links. These strong cross-linking constraints facilitated modeling of a Med17 β-ribbon that encompassed most of the cross-link hotspot (Fig. 2B). Importantly, modeling of this region of Med17 dramatically simplified the process of modeling into the remaining “Joint” density. To our knowledge, this remains a rare example of the use of cross-link constraints to interpret X-ray crystallography maps and highlights the power of integrative structural approaches to overcome the weaknesses inherent in individual structural approaches.

### 3.3 Unique architectural roles of Med17 and Med14 in the Mediator complex

The last few years have seen huge gains in our understanding of the organisation and molecular structure of the Mediator complex, both as an independent complex and also when engaged with other components of the transcriptional apparatus. Aside from the Head module and a few isolated domains from the Middle [61], [62] and Tail modules [43], [63], until very recently only a basic view of the organisation and subunit architecture of the 21-subunit core Mediator complex was available. Studies probing subunit interaction networks [49], [64], [65] as well as subunit co-expression [53], [54], [66], [67] and subcomplex isolation studies [68], [69] combined to determine the subunit composition of the Mediator modules. This data was augmented by EM studies using different labelling strategies to coarsely map the termini of various Mediator subunits onto two-dimensional projection views of the complex viewed in negative stain [70], [71]. However, until very recently no three-dimensional architectural information was available for the Middle and Tail modules, together accounting for three quarters of the mass of the Mediator complex.

The transition from coarse two-dimensional subunit mapping to the first full three-dimensional Mediator model resulted from the application of an ISD strategy to the structure of the RNAPII Holoenzyme, the complex of Mediator and RNAPII. The Holoenzyme complex is not only a functionally relevant form of Mediator, but is also more soluble and stable than free Mediator alone under the conditions used for cross-linking experiments. Using BS3 cross-linking, about 400 cross-links were identified for the Holoenzyme. Despite the availability of a low-resolution Mediator EM map [70], it was impossible to generate a model of the Holoenzyme using the cross-links without a complete Holoenzyme EM map, or a model of the Mediator using the EM map without the cross-links. As a consequence, we envisioned an ISD strategy for the isolated Mediator complex based on the transferability and the modularity principles described above. First, Holoenzyme cross-links were transferred to the Mediator-alone structural determination, guided by the low-resolution map. Second, we enforced the modularity principle, by segmenting the Mediator EM density map into regions corresponding to the Head, Middle, and Tail modules of Mediator (Fig. 4).

The Mediator model generated by this ISD approach positioned each subunit within the low-resolution Mediator envelope and provided an unambiguous picture of the organisation of the Mediator Middle and Tail modules (Fig. 2A) [17]. As well as global architectural details, the approach provided a structural framework for interpreting the role played by individual Mediator subunits in the complex. Central to understanding the organisation of the Mediator complex was shedding light on the role of the two large and essential subunits Med14 and Med17. Originally identified in a genetic screen in yeast for genes required for glucose repression [72], Med14 had been classified as a member of both the Middle and Tail modules. Furthermore, truncation of the C-terminal portion of Med14 was shown to destabilise the interaction between these same Mediator modules [73]. Reconstitution of a stable and functionally active human core Mediator complex (Head and Middle subunits) was shown to depend on the presence of Med14, suggesting an important scaffolding role within the complex [74]. By providing the three-dimensional subunit architecture of the Mediator complex, the ISD approach was the first to shed light on the structural basis of the unique scaffolding role played by Med14 (Fig. 2A). Subunit density for Med14 was mapped over a vast portion of the Mediator complex, spanning a total distance of 220 Å. Cross-linking patterns show that the N-terminus of Med14 is positioned to interact with Med10 and 19 at one extreme of the Mediator Middle module whilst central regions of Med14 encompass the remainder of the Middle. The cross-linking pattern of the C-terminal portion of Med14 showed a distinctive pattern characterised by colocalization with the Tail module proteins Med15 and 16. This observation helped to localise the junction between the Mediator Middle and Tail modules and hence explained the loss of the Tail module in Mediator preparations harbouring a truncation of the C-terminal portion of Med14 [73]. The proximity of Med14 to subunits from all three Mediator modules provided strong support to the view that Med14 plays an essential architectural role in the complex, helping to stabilise the intermodular interactions that hold the complex together. Recent high-resolution structural studies have since confirmed the localisation of Med14 first provided by integrative modeling and have provided further structural evidence for the role of Med14 to bridge the Head, Middle and Tail modules [75], [76], [77].

Likewise, Med17, the largest subunit in the Head module had been shown to act as a scaffold by forming extensive interactions with nearly all of the remaining Head module subunits. Despite playing an essential functional role in yeast (P. Robinson unpublished results), the first 181 N-terminal residues of Med17 were unstructured and unresolved in X-ray studies of the Mediator Head module [34], [58], [78]. The ISD approach provided an explanation for this missing Med17 crystallographic density, which was found to play a critical architectural role in the Mediator complex. The Med17 N-terminal domain (NTD) was found to extend away from the surface of the Head module to interact closely with a central region of the Mediator Middle module (Fig. 2A). The Med17 NTD was mapped to this region through cross-links to the Middle module subunits Med 7, 21, 4, 9, 14, and could be localised with a precision of roughly 10 Å. By extending away from the Head module to take part in an extensive interaction network with these Middle module subunits, the Med17 NTD was predicted to play an important bridging role between these two distinct Mediator modules. As with Med14, later high-resolution studies confirmed the unique bridging interactions played by Med17 within the Mediator complex [75], [76], [77] and demonstrated that the ISD approach provided an accurate low-resolution model for the N-terminal region of Med17 within the Mediator Middle module guided by a collection of CLMS distance restraints.

### 3.4 Middle module architecture

The fact that over half of the subunits comprising the 9-subunit, ∼300KDa Middle module are essential for yeast survival corroborates the idea that this Mediator region plays a central role in gene activation. As for the conserved Head module, attempts to understand the structural basis of its core biological role were initiated through the purification and X-ray crystal structure solution of small stable domains such as the Med7N-Med31 and Med7C-Med21 heterodimers [61], [62]. However, these models accounted for only a small fraction of the primary sequence of the Middle module and in isolation they provided little biological insight. Attempts to build a more complete picture of the architecture of the Middle module came first from cross-linking mass-spectrometry [79] and later from affinity labelling studies where subunits were coarsely mapped onto 2D negative-stain EM images of Mediator using labelled antibodies or based upon difference maps following subunit deletion [70], [71]. Although both of these experimental approaches provided valuable new information, neither were sufficient to provide a complete 3D architectural view of the Middle module. These studies had, however, made some interesting predictions as to the arrangement of Middle module subunits. Based upon: (1) cross-link constraints, (2) evolutionary relationships between the Med7-21 and Med4-9 heterodimers, and (3) end-to-end helical packing interactions observed within crystals of Med7C-Med21, Lariviere and colleagues predicted that an extended arrangement of end-to-end stacked helical bundles would form a central scaffold within the Middle module [79]. Later EM observations corroborated the idea of an extended Middle module architecture but were unable to resolve the internal subunit organisation [70], [71]. Although tantalizing, these early modeling efforts were manually derived, speculative in nature and remained unchallenged by rigorous computational validation procedures.

A first complete 3D architectural representation of the Middle module was produced using ISD (Fig. 2A) [17]. Crucially, this approach differed from earlier attempts to define Middle module architecture by being able to combine all available structural information, comprising atomic models, comparative models, cross-link restraints and a low-resolution EM envelope in an unbiased computational approach to exhaustively sample configurational space. Strikingly, the computational approach confirmed that the elongated Middle module density was defined by an end-to-end packing of the 4-helix Med7C-21 and Med4N-9 heterodimers. This central scaffold was localised within the EM density at a high level of precision, with an average root means square fluctuation (RMSF) of around 10 Å within the cluster of top-scoring model solutions. The architectural model provided details of the arrangement of Med10 and Med19 at one extreme of the elongated Middle module and their binding interface with the 4-helix bundle of Med7C-21. At the other extreme the large subunits Med14 and Med1 were shown to interact on opposite surfaces of the equivalent helical bundle formed by Med4N-9. As described above, the model also provided details of an extensive network of interactions positioned close to the central Med7C-21/Med4N-9 junction that involves not only these four subunits but also the N-terminal extension of the Head module subunit Med17 and central portions of Med14. Such details provided by the architectural model of the Middle module produced by integrative modeling have since been validated by high resolution structural studies using X-ray crystallography and cryo-EM (Fig. 2A) [75], [76], [77]. Such validation confirms that integrative modeling techniques can produce a very accurate description of the internal 3D organisation of multi-subunit assemblies and form a strong foundation from which to design further structural and functional analyses.

### 3.5 TFIIK location in the pre-initiation complex

TIIK is a trimeric subassembly within the large multi-subunit general transcription factor TFIIH. Whereas the remainder of TFIIH functions as an ATP-dependent DNA helicase/translocase with roles in promoter DNA opening [80] and DNA damage repair [81], the TFIIK trimer performs a disparate role as a protein kinase. Yeast TFIIK is composed of the cyclin-dependent kinase Kin28, its cyclin Ccl1 and a third protein Tfb3, which together phosphorylate the C-terminal domain of the largest RNAPII subunit Rpb1 [82]. Phosphorylation of Serine 5 residues within the highly conserved tandem CTD heptapeptide repeats correlates with disruption of the Mediator-RNAPII interaction at the gene promoter [37] and transition of RNAPII to the elongation phase of the transcription cycle [38], [39]. Therefore, in the context of the promoter-associated pre-initiation complex, TFIIH simultaneously makes two important contributions to transcriptional initiation: (1) ATP-dependent DNA translocase activity to melt the duplex promoter DNA and allow engagement of the template strand in the active cleft of RNAPII and (2) destabilisation of the PIC through CTD phosphorylation leading to RNAPII promoter escape. Understanding the structural basis of these two functions and how they are coordinated in the context of the pre-initiation complex has been a key focus of a number of recent structural studies. The architecture of the 31-subunit PIC, lacking Mediator, was first determined using *in vitro* reconstitution and cryo-EM analysis of the human [83], and subsequently the yeast [84] promoter assemblies. These studies demonstrated that the PIC has a bilobal structure with one lobe comprised of RNAPII with a number of the general transcription factors including TBP, TFIIA, -B, and -F and the second comprised of TFIIH and regions of TFIIE. In these studies, promoter DNA could be traced through the structure, with large distortions introduced through interactions with TFIIB and TBP, such that prior to DNA melting, the double stranded DNA is directed along the active site cleft of RNAPII before exiting along a path that allows downstream contacts with the Ssl2 translocase subunit of TFIIH. Negative stain EM with affinity labelling [85] as well as ISD analyses [16] had previously determined the course arrangement of subunits within the yeast TFIIH complex. These analyses suggested that the two TFIIH ATPase subunits Ssl2 and Rad3 are associated at opposite ends of a central multi-subunit core (comprising Tfb1/2/4/5 & Ssl1), with the TFIIK trimer at a more peripheral location, adjacent to Rad3. This prior information helped to interpret TFIIH density in the PIC cryo-EM maps and position the core and associated Ssl2 and Rad3 ATPase subunits. Ssl2, the TFIIH translocase with a prominent role in transcriptional initiation, was positioned to interact with the downstream promoter DNA, and at the other end of the TFIIH core a Rad3 homology model could be unambiguously docked into corresponding EM density [84]. However, no density was found corresponding to the ∼120KDa TFIIK trimer in the vicinity of Rad3 or otherwise, and therefore TFIIK was assumed to be highly flexible and averaged out during image processing.

The location of TFIIK within the PIC was determined using an integrative structural biology approach in which cryo-EM was combined with CLMS and ISD to calculate and interpret EM density for an *in vitro*-assembled 52-protein Mediator-bound pre-initiation complex (Fig. 2C) [18]. Mediator and PIC EM densities could be docked into the Med-PIC map with little evidence of structural perturbation upon complex formation (Fig. 2A). However, such docking highlighted a region of PIC density immediately adjacent to Rad3 that had not been observed in the analysis of PIC complexes lacking Mediator. As the Mediator complex had earlier been shown to stimulate the CTD kinase activity of TFIIK [27], and since TFIIK was missing in the earlier PIC reconstructions [83], [84], the TFIIK trimer seemed an obvious candidate for the extra PIC density, which appeared to become ordered upon Mediator binding. An ISD approach was used to test whether this extra density, segmented from the Med-PIC EM map, was consistent with TFIIK cross-link restraints (Fig. 2D). The analysis was performed by fixing the adjacent Rad3 homology model at its corresponding position in the Med-PIC EM map as an anchor and then searching configuration space for solutions for TFIIK subunit localisations that best satisfy both TFIIK cross-links and EM density restraints. Importantly, all top scoring models from these trial simulations represented a single self-consistent TFIIK subunit architecture that localised to the extra EM density whilst fully satisfying TFIIK cross-links. The architectural solution demonstrated that direct interactions between Rad3 and Tfb3 likely underpin the connectivity of TFIIK to the TFIIH core whilst proximity to subunits in the Mediator Middle and Head modules likely play a role in positioning TFIIK in space. Localising TFIIK within the PIC provided a clear explanation of the role of Mediator in stimulating the CTD kinase activity of TFIIK. Unbiased docking of the Head-CTD crystal structure within the PIC shows that Head interactions position the CTD in the immediate vicinity of TFIIK. Therefore, the structural evidence strongly suggests that Mediator acts to simultaneously position both the RNAPII CTD and TFIIK trimer within the PIC and bring them into close proximity in order to stimulate CTD kinase activity.

### 3.6 Tail module architecture, dynamics and activator interactions

The Tail module is composed of non-essential protein subunits with higher levels of sequence divergence than observed within the Head and Middle modules. Rather than contributing to the basal transcription initiation mechanism the Tail module is instead responsible for mediating the regulatory response to sequence-specific DNA-binding transcription factors [66]. Indeed, the yeast Tail module Med2-Med3-Med15 triad has been shown to be a common target of transcriptional activator proteins including the extensively studied Gal4 and Gcn4 [68], [86]. Studies exploring the nature of the Gcn4-Mediator interaction have described a “fuzzy” binding mechanism whereby numerous low-specificity and low-affinity hydrophobic interactions function additively to amplify the overall binding affinity between activator and Mediator [43]. A recent study combining cross-linking with NMR demonstrated that interactions between the Gcn4 activation domains (ADs) and multiple activator-binding domains (ABDs) within Med15 are very heterogenous in nature with all possible AD-ABD combinations detected and contributing to a large “fuzzy” interaction surface [44]. The generality of this binding mechanism is still an open question but it is interesting to note that transcriptional coactivators often harbour multiple ABDs, and such a model could potentially explain how a collection of otherwise unrelated transcriptional activator proteins can converge on a limited number of transcriptional coactivator targets. A further open question is how the transcriptional response is modulated by transcription factor-Mediator interactions. Do such interactions simply increase the residency of Med-PIC complexes at the gene promoter, as some have suggested [87], or does transcription factor binding have an allosteric effect, leading to conformational changes that modulate transcription levels? Evidence for the latter has come from EM studies in which activator binding events have been correlated with structural changes within the Mediator complex [88]. To date, the Tail module remains the least well characterised of the three Mediator modules. For example, high-resolution structural studies of the Tail have been limited to a modest collection of small protein domains [43], [89]. Low-resolution mapping of subunit termini within EM projection images provided some insight into the relative location of the various Tail subunits, but this information was restricted to two dimensions [70], [71].

**Fig. 3:**
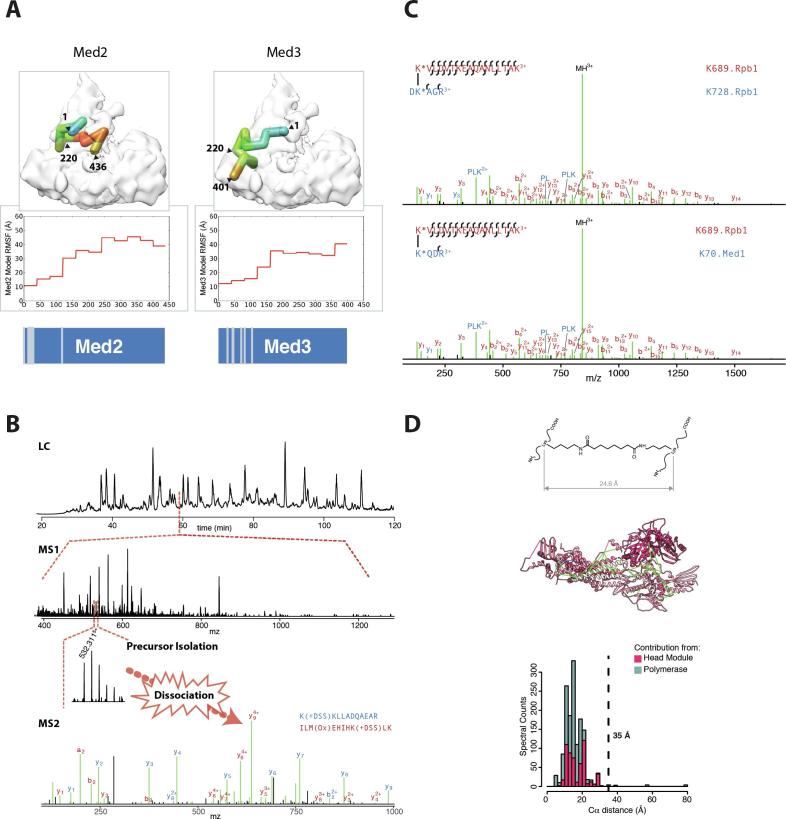
Cross-linking MS guided integrative structure determination. (A) Cross-linking coverage plays a key role in determining how precisely a model component can be localized. Med2 and Med3 both have numerous inter-protein cross-links constraining their N-terminal regions in the Mediator model (represented on bottom panel by grey lines on blue schematics), yet none were found after the first 130 residues. Coarse grained beads representing 40 residues of Med 2 and 3 are localized in the final model with RMSF precision between 10 and 20 Å from the N-terminal region (blue shades in upper heat map representation). Beyond this, the precision rapidly deteriorates (red regions of heat map). (B) Cross-linked peptides can only be measured by a mass spectrometer if the precursor ion is successfully selected for dissociation and product ion analysis. Tryptic digests of large, purified protein assemblies contain hundreds of peptide ions co-eluting from the nano-LC column at any given moment even after fractionation to enrich for cross-linked peptides. The upper panel shows the total ion current corresponding to a single fraction of a size exclusion chromatography-based cross-link enrichment. The precursor ion scan (MS1) taken at 59 min shows hundreds of ion signals co-eluting. Cross-link identification requires isolation of the quintuple charged precursor ion at 532.31 *m*/*z* for gas phase dissociation and measurement of the product ion (MS2) spectrum. Factors influencing whether the cross-link will be identified include: the scan rate of the mass spectrometer, the extent of enrichment/fractionation in the 1st- chromatographic dimension, the peak capacity and gradient length in the 2nd-chromatographic dimension, and the ionization efficiency of the cross-linked peptides and their intensity relative to co-eluting peptides. (C) After measuring a cross-linked product ion spectrum, successful identification requires sufficient product ion formation to identify both members of the cross-linked peptide pair. The upper and lower panel show the best and second-best hits to a cross-linked product ion spectrum after a database search. The correct hit, corresponding to an intra-protein cross-link on Rpb1, differs from the incorrect hit, a potentially more interesting inter-modular cross-link between Rpb1 and Med1, by a single blue y3-ion. In both cases, almost all of the product ion signals are matched to the sequence (green lines), with the longer, red peptide accounting for most of these. (D) Mapping cross-links to existing high-resolution structure provides one method of validating a dataset. Common reagents such as BS3 and DSS leave a suberate bridge between adducted lysine residues. To account for the flexibility of the lysine side-chain, distances are measured between Cαs, giving an expected span of about 25 Å. In practice, 30 or 35 Å is typically used as the violation distance to account for protein dynamics and uncertainty in the structural coordinates. (For interpretation of the references to colour in this figure legend, the reader is referred to the web version of this article.)

The first 3D architectural details of the Tail module were provided alongside those for the Middle module in an ISD study describing the subunit architecture of the full Mediator complex [17]. The model that resulted from this study localised each Tail module subunit within the 3D EM envelope and provided a structural explanation for the EM labelling studies, protein-protein interaction data and sub-complex isolation observations reported previously [49], [54], [64], [65], [67], [68], [69]. In the case of the Tail module, the architectural solution was strongly influenced by the inclusion of a 540-residue homology model for the N-terminal portion of Med16, identified by strong sequence similarities to the 7-bladed β-propeller of the *S. cerevisiae* vesicle coat protein Sec31. Positioning of this homology model through cross-linking and EM constraints helped to determine the proper location of all other Tail module components. Specifically, Med2, Med3 and Med15 that make up a Tail module triad commonly targeted by activators, were found to colocalize with the C-terminal region of Med14 at the junction between the Tail and Middle modules. The N-terminal portions of Med2 and Med3, both of which are strongly predicted to form a coiled-coil motif, were colocalized with particularly high precision to a single region of the Tail (Fig. 3A), supporting the idea of coiled-coil interactions and explaining the observation of Med2-3-15 sub-complex isolation in earlier biochemical studies [68]. Likewise, the observed Med16 β-propeller-Med5 interface explained earlier reports of Med5-Med16 N-term sub-complex isolation [70].

**Fig. 4:**
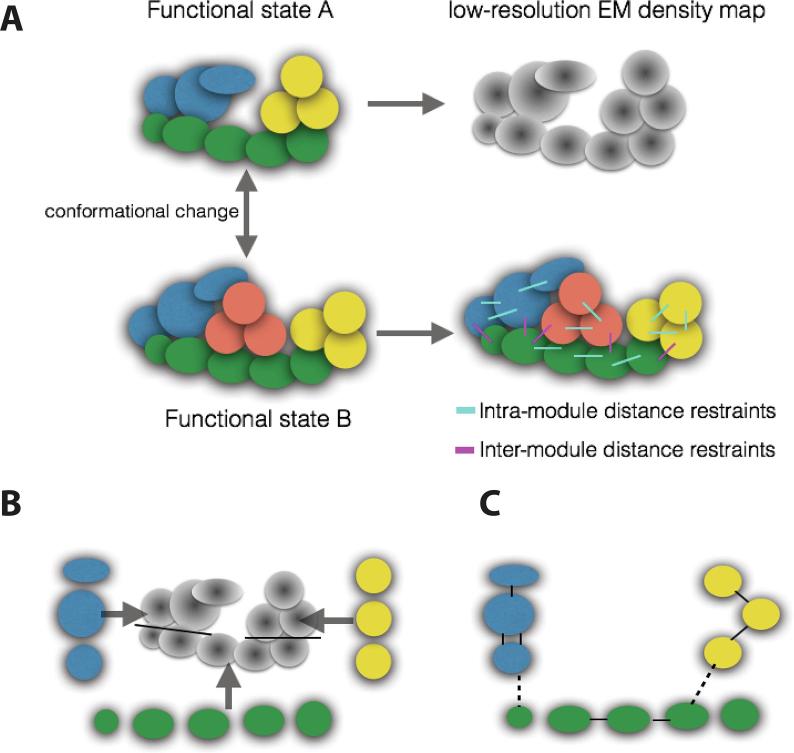
Exploiting structural modularity and data transferability. The assembly is a stable aggregate of individual macromolecules (circles), organized into modules (blue, yellow, green and coral colours). (A) The architectures of two functional states of the same assembly are related by a global conformational change which rearrange the modules without significant perturbation of their internal structure. The two functional states are for instance obtained by varying the composition of the two samples. Functional state A is used to derive a low-resolution 3D density map, by cryo-EM experiments (grey circles), while functional state B is utilized to collect CLMS data, which can be grouped into intra- and inter-module cross-links (coloured lines). (B) If the target of the modeling is the structural determination of the functional state A, the different proteins and components belonging to the modules are attributed to the corresponding regions of the 3D density map, after a suitable segmentation of the density (black lines). (C) To allow the violation of the inter-module distance restraints, two different unknown weights are attributed to the inter- and intra-module cross-links (dashed and continuous lines, respectively), which were collected for functional state B and transferred to functional state A. The weights are determined during the modeling, using the Bayesian scoring function. (For interpretation of the references to colour in this figure legend, the reader is referred to the web version of this article.)

Information on the architecture and dynamics of the Tail module was expanded in the integrative structural studies of Med-PIC, which revealed some surprising novel features of Tail module biology (Fig. 2E). Firstly, all prior structural studies on the Mediator complex had suggested that only a single inter-module connection existed between the Middle and Tail modules, for which the C-terminal domain of Med14 was shown to be a critical component [70], [71]. However, evidence from cryo-EM and cross-linking on Mediator-bound to the PIC showed that in fact a second Middle-Tail connection existed and was brought about by a direct interaction between Med1 and Med5 from the Middle and Tail modules, respectively (Fig. 2E). Such a direct interaction had been picked up in yeast two-hybrid screens but overlooked due to a lack of supporting structural evidence [49]. Second, the Tail module was observed in two distinct structural states. The most populated and best resolved state, termed the “Tail Down” state (Fig. 2C) has the Med2-Med3-Med15 triad positioned a significant distance from the upstream transcriptional activator sequences and out of range to form cross-links to other components of the transcriptional apparatus. However, a second lower populated state, termed the “Tail Up” state, involves a 50 Å movement of the whole ∼450KDa Tail module toward the upstream promoter DNA bringing the Tail in close proximity to the back side of RNAPII (Fig. 2E). Cross-linking analysis on Med-PIC, which in contrast to the cryo-EM, was conducted in the presence of the transcriptional activator Gcn4, corroborated EM observations of a “Tail Up” conformation with numerous Tail-RNAPII and Tail-Gcn4 cross-links observed in the CLMS dataset. Such cross-links were only consistent with a Tail module that had transitioned from the “Tail-down” to “Tail-up” conformations, which raised the tantalising possibility that activator interactions may be involved in stabilising such a structural transition. A comparison of the Tail-Gcn4 cross-link pattern observed either within the Med-PIC complex or for isolated Gcn4 and Med15 domains, highlights some interesting points about the mechanism of Gcn4 activation at gene promoters. The Gcn4-Med15 interaction is driven by hydrophobic interactions between residues in the intrinsically disordered N-terminal activation domain of Gcn4 and hydrophobic surfaces found within four Med15 regions, an N-terminal KIX domain and three ABDs (Fig. 2E) [90]. Biochemical studies have scrutinised these interactions at the primary sequence level [91] and a solution structure of Med15 ABD1 bound by residues from the Gcn4 activation domain demonstrated that Gcn4 residues adopt a helical conformation upon binding to allow aromatic residues to insert into a shallow hydrophobic Med15 cleft [43]. Interactions between residues within the Gcn4 activation domain and Med15 KIX domain and ABDs, have been consistently observed both in the context of the Med-PIC complex as well as more simple systems limited to just the Gcn4 activation domain and truncated Med15 constructs [44]. However, cross-link analysis within the Med-PIC assembly portrays a more complex picture for Gcn4-Mediator interactions. Specifically, the Gcn4-Mediator cross-link with the highest overall spectral count was not between a Gcn4 residue within the N-terminal activation domain, but instead between a C-terminal Gcn4 residue located at the terminus of the DNA-binding bZIP domain. Importantly, within Mediator this cross-link mapped to Med15 ABD2, which was also found to be heavily cross-linked to Gcn4 residues within the N-terminal activation domain, as expected. Indeed, a striking picture emerged where residues from both the Gcn4 activation and bZIP domains formed an overlapping pattern of cross-links within the KIX and ABDs of Med15 (Fig. 2E). No such pattern has been observed in studies probing the interaction of defined Gcn4 and Med15 domains simply because the Gcn4 bZIP domain was not present [44]. One intriguing interpretation is that the Gcn4 activation and bZIP domains are colocalised at the Med15 binding interface, which could only result from some unexpected extension of the Gcn4 N-terminal activation domain away from the DNA towards the C-terminal end of the bZIP coiled-coil domain. Further structural studies of Med-PIC with Gcn4 bound upstream will be required to further address such mechanistic questions. Although the “fuzzy” nature of the Gcn4-Med15 interface would make high-resolution studies of Gcn4-Tail interactions extremely challenging, many important questions could be addressed by visualising the interaction even at more modest resolutions. For example, such reconstructions could help address whether bending of the upstream DNA is required to bring bound activators in range of interactions with the Tail module. Even in the “Tail-Up” state, Med15 was found to be spaced roughly 80 Å from the site of Gcn4 DNA binding meaning that DNA bending or further Tail motion could well be involved in bringing Gcn4 and Med15 together.

## 4 Practical approaches to integrative modeling of the transcription initiation machinery

### 4.1 Cross-link mass spectrometry and the modularity of the proteome

While integrative structure determination (ISD) aims to utilize as much experimental data as possible, in recent practice, CLMS, alongside cryo-EM, has become one of the pillars on which structural studies of multiprotein assemblies rely. Bifunctional molecules are reacted with a protein complex and the sites of adduction in the resulting peptides are characterized by mass spectrometry [92], [93], [94]. CLMS can generate thousands of pairwise distance relationships (“cross-links”) from purified macromolecular assemblies, typically requiring 10–200 µg of starting protein complex. The current popularity of CLMS comes from the development in the last 10 years of robust experimental protocols to enrich cross-linked peptides [95], [96], more sensitive mass spectrometers with higher resolving power and faster scan rates [97], bioinformatic algorithms capable of addressing the quadratic increase in search space in large-scale cross-linking experiments and reliably estimating error rates [98], [99], [100], and scoring functions to encode cross-link derived spatial restraints and evaluate them against three-dimensional structural models [101]. CLMS experiments can generally be performed on the same sample preparations used in cryo-EM and do not require any additional genetic manipulations or isotopic labelling that might be necessary for FRET, NMR, or proximity labelling MS, alternative experimental techniques to provide pairwise distance constraints at various resolutions. Recent developments make use of gas-phase cleavable cross-linking reagents in conjunction with multiple rounds of ion isolation and dissociation in the mass spectrometer [102], [103] to perform CLMS experiments on cellular or organelle level preparations [104], [105]. Hence, our ability to identify cross-links confidently by mass spectrometry is not limited by the complexity of the sample preparation, although deriving meaningful structural models from extremely heterogenous mixtures, such as a nuclear preparation [106], remains conceptually unaddressed. A complete review [92], [93], [94] or protocol [107] for performing and analysing CLMS experiments is outside the scope of this manuscript, and has been covered elsewhere. This section instead will focus on sometimes overlooked nuances and considerations involved in preparing cross-linking datasets for ISD.

Cellular organization is modular and hierarchical [108], [109]. Hence, individual polypeptide chains are frequently found bound together only in highly stable complexes. At the next level of organization, these core complexes interact with other core complexes in more dynamically regulated ways to assemble functional protein machines. Protein machines interact with each other through ever more transient and fleeting interactions. Transcriptional initiation perfectly illustrates this premise in the way that stable core modules, such as Mediator Head, Middle, Tail, RNAPII, and TFIIK must all physically interact in a coordinated fashion to convey the action of a gene activator binding at an enhancer into a regulatory signal initiating the escape of pol II from the promoter and the onset of mRNA synthesis. This modularity has several implications for CLMS experiments. First, giant macromolecular targets can be built up in a bottom-up fashion from experiments on smaller core complexes. For instance, our work on the Med-PIC, made use of and integrated all previously published CLMS experiments on many of its subcomponents, including TFIIH [16], core initiation complex [110], PIC [111], and our work on the Mediator [17], to assemble a combined CLMS dataset of ∼2500 unique cross-links. In many cases, the structures of the core modules will remain fixed, with conformational changes taking place at the interface of the modules. Hence, it is useful to distinguish between intra-modular and inter-modular cross-link restraints, as their transferability to other systems can reasonably be expected to differ based on this classification. An example of how inter-modular and intra-modular cross-link restraints were scored differently in the Mediator complex modeling is given below (Section 4.5).

Secondly, higher order interactions at the interface of two modules are by definition, more transient, and hence more difficult to capture by covalent cross-linking approaches. For instance, we’ve observed that inter-modular cross-links comprise only 8–18% of the total data in the Med-PIC experiments. This is of course, unfortunate, since inter-modular cross-links tend to be the most novel and important in terms of elucidating new structural biology. One route towards improving the yield of inter-modular cross-links might be in using cross-linking reagents with quicker labelling kinetics that can capture more transient states of the assembly. Typical cross-linkers are activated esters, which acylate protein-based nucleophiles, such as the ε–amino group of lysine. The kinetics of the acylation reaction can be modulated by changing the leaving group of the cross-linking reagent. For instance, changing the leaving group of the commonly used cross-linking reagent disuccinimidyl suberate (DSS) from N-hydroxy-succinimide to 1-hydroxy-7-azabenzotriazole in the SBAT reagent, increases the rate of reaction seven-fold and additionally produces a greater ratio of cross-links to non-productive dead-end modified peptides [112]. Another approach to increasing the yield of inter-molecular cross-links is to employ a two-step cross-linking strategy. Substoichiometric formaldehyde is first applied to fix the system in a reversible cross-linking step, followed by cross-linking with an irreversible reagent such as DSS or SBAT. The formaldehyde fixation is reversed during work up, similar to chromatin immunoprecipitation (CHIP), and the remaining cross-links analysed as usual. Our pilot experiments with the Holoenzyme and Med-PIC complexes showed a 10–50% increase in the number of cross-links when preceded by a formaldehyde pre-fixation step (Trnka – unpublished results), although these increases were not limited to inter-modular cross-links and the effect has not been fully explored.

### 4.2 Which Residue-residue contacts are experimentally observable by cross-linking mass spectrometry?

While CLMS is occasionally referred to inaccurately as an unbiased sampling of distance restraints, in reality a number of factors influence the sparseness of cross-linking data sets and whether a cross-link is detectable by mass spectrometry? In order to observe a cross-link, (1) a pair of reactive residues must reside in the protein assembly within: the geometric span that is accessible to the cross-linking reagent, a temporal span consistent with the kinetics of covalent capture, and a local chemical environment that is favourable to the conjugation reaction, (2) each of the container peptides must have physio-chemical properties that are amenable for mass spectrometry analysis, (3) the precursor ion of the cross-linked peptide must have enough signal intensity for it to be selected for dissociation by data-dependent MS sampling schemes, (4) each of the cross-linked peptides must produce sufficient product ions to unambiguously assign their identity.

There are an endless number of variations that can be made to the chemistry of cross-linking reagents to address the first consideration. For instance, cross-linking molecules can be designed with varying bridge lengths, reaction kinetics, membrane permeability, or target specificity [113]. However, there are some inherent limits imposed by the protein assembly itself. The target amino acids must also be solvent accessible and available for reaction. The reactivity of a lysine residue targeted by a typical NHS-ester reagent is dependent on hydrogen bonding and the local pH at that site. There is a wide range in a lysine’s inherent reactivity depending on the extent to which it is protonated or hydrogen bonded and it is common to see that cross-links are concentrated at a smaller number of highly reactive lysines that cross-link to multiple other sites. Modulating the amino acid specificity of a cross-linking reagent to target acidic residues [114] provides one route around this issue, although this can considerably complicate the ability to localize the cross-link to a particular residue based on its product ions (“site-assignment”) as well as increasing the complexity of the reaction mixture and lowering the cross-linking yield. The secondary structure of the target protein influences the outcome of cross-linking as well, with cross-links most likely to occur at loops and helices with fewer cross-links observed from beta-sheeted regions. For our Med-PIC study, comparing the distribution of predicted secondary structure at the adducted residues against that of all residues shows helical cross-links as 121% the expected amount, loop cross-links at 97% expected, and sheeted cross-links at 41% expected.

Secondly, certain peptides are more likely to be measured during a typical proteomics experiment. Peptides with length between approximately 5 and 30 residues and bearing some positive charge are ideally detected by reverse-phase chromatography coupled online to positive mode electrospray ionization and fragmented by collisional dissociation. Hence, trypsin has always been the ideal protease for proteomics experiments as the distribution of lysine and arginine residues generates favourable peptides. The use of other enzymes, such as chymotrypsin, Asp-N, or Glu-C may provide some complementary cross-link coverage for difficult to access regions, but in general these enzymes do not provide such favourable peptides for mass spectrometry analysis and comparative studies have not seen much benefit [96]. Namely, these enzymes tend to create peptides that are too short and are hard to identify unambiguously, or else peptides with little positive charge that do not ionize well. There is additionally a nice interplay between trypsin selectivity and lysine-directed cross-linking, in that modification at lysine by the cross-linking molecule blocks trypsin cleavage, ensuring that most cross-linked peptides will only have one likely site of modification. A mass spectrometer can also be tuned for peptides that do not match the criteria listed above. For instance negative ion mode can be used for acidic peptides, or mobile phases can be altered to favour hydrophobic or membrane spanning peptides. In practice, most mass spectrometry facilities are highly oversubscribed and changing the operating procedures of a major instrument places a burden on the available resources.

Third, MS-proteomics are typically conducted in data-dependent acquisition (DDA) mode. There are typically many more peptide ions eluting at a given time from the LC-column than can be isolated, dissociated, and measured (Fig. 3B). Therefore, DDA mode programs the instrument to select ions based on certain criteria, such as ion intensity and charge state. In some cases, DDA mode can select for the presence of a pattern of ions that is characteristic for cross-linked peptides, such as a pair of ions separated by a defined mass difference due to the use of isotopically heavy and light versions of a cross-linking reagent [115]. Of the four aspects required to detect a cross-link, this is the most easily modified or optimized. Cross-linked peptides can be enriched by size-exclusion chromatography [96], cation exchange [95], or by affinity tags in the reagent [116] or the protein complex of interest [24]. Furthermore, the reverse phase chromatography that is directly coupled to the mass spectrometer can be optimized for longer gradients using longer packed or monolithic columns [18]. DDA analysis for cross-linked peptides typically programs mass spectrometers to exclude singly and doubly charged precursors to focus on cross-linked peptides which tend to be quadruply charged or higher. Cross-linking chemistries can be employed that increase the charge state further still [117], [118] for improved targeting of cross-linked versus linear peptides. An exciting development is the recent increase in coupling of ion mobility separation (IMS) with mass spectrometry [119], [120]. IMS provides a millisecond scale separation of ions in the gas phase based on collisional cross-section and hence charge-state and promises to provide a route to further enrichment and isolation of cross-linked peptides.

Finally, cross-linked peptides, after being isolated for dissociation, must produce enough product ions such that search algorithms can identify both of the cross-linked peptides [100]. Collisional based dissociation methods produce fragment ions according to mobile proton theory [121] and whether product ions from both peptides are observed largely depends on the gas-phase basicities and charge distributions of the peptides. In cross-linking-MS, a common situation is that the product ion distribution identifies one peptide very well, but leaves the second peptide poorly identified. Gas-phase cleavable cross-linking reagents circumvent this problem by containing low-energy bonds that dissociate prior to the peptide backbone during MS2. Subsequent isolation of the individual peptide ions and a second round of dissociation in MS3 provide fragmentation information for each peptide [102], [103]. This approach provides both the individual precursor masses for each peptide as well as their fragmentation patterns and can aid in correctly identifying the peptides in the cross-link. In practice, these schemes have inherent trade-offs such as an increased mass spectrometry cycle time resulting in fewer cross-linked precursors being sequenced, as well as insufficiently specific chemistry in MS2 leading to inefficiencies in targeting the correct precursor ions for MS3. Electron-transfer dissociation (ETD) is an alternative to collisional activation which shows less specificity to the physicochemical properties of the peptide analytes [122]. Efficient electron transfer to begin the dissociation process requires positively charged peptide substrates, so that ETD should be the ideal method for sequencing of positively charged, cross-linked peptides to give an equal distribution of product ions from both peptides. This method was infrequently applied however until recently, because ETD implementation has consistently lacked in speed and sensitivity to collisional methods. However, newer implementations [123] are changing this situation and ETD is likely to become the method of choice for cross-linking analysis.

### 4.3 “Noise” in cross-linking data sets and implications for ISD

In practice, cross-links are never identified completely unambiguously. For any non-trivial-sized search space, a given spectrum will match to multiple theoretical cross-linked peptide pairs that are within the *m*/*z* tolerance of the precursor ion. These matches need to be discriminated on the basis of how well the experimentally observed product ions are explained by the theoretical cross-linked peptides. Typically, the matched product ion series are incomplete, and the experimental spectra are noisy and contain product ion signals that come from either co-eluting peptides with similar precursor *m*/*z* or otherwise cannot be explained by the cross-linked peptide sequence. Especially for short peptide sequences that are four or five residues long, multiple proteins in a database might contain either identical or isomeric versions of these peptides that differ only in the arrangement of amino acid residues. In these cases, the correct cross-linked peptide match can sometimes differ from the next best match by only a single additional product ion match (Fig. 3C).

Various metrics describing the match of the theoretical cross-linked sequence to the experimental spectrum are reported by proteomics search engines along with an overall statistically-derived classification score. Hence, like other MS proteomics datasets, cross-linking data are expected to contain misidentifications. Datasets are reported alongside a false discovery rate (FDR), which is commonly based on conducting the search against both the target protein sequences and randomized, decoy versions of the same proteins [124]. For this approach to accurately estimate the chance of a false match, a large enough decoy database must be used to reliably model the distribution of incorrect hits. Hence, the typical proteomics approach of using a decoy database equal in size to the target database is not appropriate for small database searches containing a limited number of protein sequences. In our Mediator studies, we used a decoy database that was 10x larger than the target database, making the total number of protein sequences searched ∼700.

Additional validation of cross-linking data comes from mapping the identified cross-links onto the atomic structures (when available) for components of the complex. The distance between Cα atoms of the cross-linked residues should be consistent with the length of the cross-linker bridge group plus the length of the adducted amino acid side chains. An additional 5–10 Å are typically added on top of this to account for imprecision in the atomic coordinates and dynamic motions in the protein complex. Hence BS3 or DSS cross-links are generally considered “violations” when the Cα -distance exceeds a limit of 30 or 35 Å, which includes the suberate bridge (∼12 Å), the length of two lysine side chains (∼12 Å), plus an addition 5 or 10 Å (Fig. 3D). The violation rate for a cross-linking dataset is often similar in value to the FDR. However, the FDR formally describes the proportion of reported residue pairs that are incorrectly assigned, whereas there are a number of other reasons why violations may occur. These involve deviations from the expected crystal structure either due to a conformational or configurational discrepancy between the sample being cross-linked and the structural model used for assessment. Therefore, a violation rate that is substantially higher than the FDR can indicate either a discrepancy in the conditions used to obtain the different types of structural information, or a broader problem such as protein aggregation under the cross-linking conditions.

With respect to utilizing cross-linking derived restraints in integrative modeling procedures it is important to realize that cross-linking data are expected to contain incorrect or otherwise inapplicable restraints, and that the probability of an individual restraint being incorrect is reflected in a classification score, which can be passed on to the modeling parameters. In this way, cross-links with more certain assignments can be weighted more strongly than others. One aspect of experimental mass spectrometry data that can be taken advantage of is that there might be multiple lines of evidence supporting the assignment of some cross-links. A CLMS experiment generates between thousands and hundreds of thousands of product ion spectra that are all searched for potential cross-links. Spectra that match above some classification threshold are termed Cross-linked Spectral Matches (CSMs). CSMs match a cross-linked peptide pair, and the cross-linked peptide pair identifies a cross-linked residue pair (“cross-link”). Hence a given cross-link is often identified by multiple cross-linked peptide pairs, which are identified by multiple CSMs. It is important to note that this redundancy derives not only from picking the same precursor multiple times over one or more experiments, but from redundancy in the chemical entity identifying the cross-linked residues. For instance, cross-linked peptides can be identified from differently charged precursor ions, or one or both of the peptides might differ with respect to the length of the peptide (due to missed enzymatic cleavage), with oxidation state at methionine residues, or due to the presence of a “dead-end” modification (in addition to a missed cleavage). These factors will produce different precursor ions at different *m*/*z* values with different product ion spectra. Hence, cross-links identified from multiple cross-linked peptides have multiple lines of evidence pointing to their existence. Cross-links identified from multiple CSMs however, are not necessarily more confidently identified as the search algorithms are deterministic so that incorrectly identified CSMs will be identified consistently leading to redundant incorrect CSMs.

### 4.4 Treatment of the input data for ISD

The first stage of the ISD approach consists of gathering all the available data needed to build the model and validate it. Spatial information about a given system can include data from a variety of experiments, statistical propensities extracted from known homolog sequences or protein structures, and physical laws, such as atomic interactions obtained from a molecular mechanics force field [46]. In our approach input data include the sequences of the subunits and their stoichiometry, the available atomic structures of domains, subunits or subcomplexes, CLMS data with the identification frequency and identification scores of the cross-links, low-resolution cryo-EM density maps of the complex, EM-labeling or EM-deletion experiments, and protein-protein interactions from affinity purification and yeast two-hybrid assays. All the input information is used to build the representation, the scoring function, and the validation toolbox for the system. First, crystallographic structures of the subunits and homology models are used to build the representation. Second, the cross-linking data and the EM density data, the sequence connectivity and physio-chemical data are used to build the scoring function, third, data which are difficult to encode in a scoring function are left out of the modeling procedure and used for validating the final model.

For integrative modeling of the 21-subunit Mediator complex [17], which will form the basis of discussions in Sections 4.4–4.9, the data included:

(1) 298 Mediator cross-links identified from 1900 spectra, consisting of multiple peptide sequences, charge states, or replicates.
(2) The atomic structures, including X-ray crystallography structures and homology models, covering 80% of the Mediator Head module, and overall 23% of the residues in the Mediator complex. It is important to note that whilst the resolution of the output models is influenced by the coverage of atomic structures, a large percentage of atomic structure is not required to produce a descriptive ISD model. Early ISD models for the nuclear pore complex did not contain any atomic structures [125] and the coverage for the Mediator [17] and SEA complexes (35% coverage) [126] were both very low.
(3) An 18 Å resolution cryo-EM density map of the Mediator complex was used to constrain the overall-shape of the complex.
(4) Validation data, such as yeast two-hybrid, immuno-precipitation, subcomplex isolation assays, and protein localization from labeling and domain deletion EM studies. The validation data was not directly used in the modeling, but *a posteriori*, to assess the quality of the models.

All the modelling protocols were scripted using the Python Modelling Interface (https://github.com/salilab/pmi) [15], a library to model macromolecular complexes based on the open source IMP package (http://salilab.org/imp/) [46]. Files containing the input data, scripts, and output models are available at a free repository, see for instance http://salilab.org/mediator and https://zenodo.org/record/802915.

### 4.5 System representation and scoring function development for ISD

The computational representation of a macromolecular system is the collection of all the degrees of freedom that need to be determined based on input information [14]. The representation assigns the system components (e.g. residues, atoms or domains) to geometric primitives with 3D coordinates (e.g. points, spheres or 3D gaussians) needed to compute the score (Fig. 1). This assignment is decided before any other computations, and is fixed along the computation. Recently, protocols to optimize the molecular representation have been introduced [127], where the optimum is the representation with the highest resolution for which sampling is still exhaustive and the models have a precision that is commensurate with the precision of the representation. The radius of the spheres and the variance of the Gaussians were set to describe the average molecular volume and the molecular electron density of polypeptide segments, respectively. The representation also uses rigid bodies, where the relative positions of the primitives (e.g. residues in a domain) are constrained based on a crystallographic structure. We use coarse-grained beads to encode the uncertainty related to the degrees of freedom of protein regions which are not directly represented by atomistic structures in the input data. These coarse-grained beads are free to move as spheres on a string, as opposed to the rigid-bodies, and are connected based on the amino acid sequence. The resolution of the coarse graining is commensurate with the resolution of the input information, in our case the cross-links and the cryo-EM density map. As a consequence, the representation is multi-scale, where different parts of a structure are represented by different coarse-graining levels. This strategy maximizes computational efficiency while avoiding model oversimplification.

In our modeling approach we used three scales to represent the system where the rigid-bodies are defined: the first is where each bead corresponded to individual residues, and was centered at the position of the C_α_ atom, the second is where each bead represented 10-residue segments and was positioned on the center of mass of all atoms of the corresponding segment, and the third scale is the Gaussian mixture model (GMM) approximation of the electron density of the corresponding structure [128]. We adopted a two-scale representation for the flexible strings: the first is where each bead represented either a 20- or 40-residue segment and was positioned on the center of mass of all atoms of the corresponding segment. The second is a spherical Gaussian. In this case the bead and the Gaussian centers were enforced to be identical.

The gathered data and the representation are next used to build a scoring function, which ranks alternative models based on how well they quantitatively reproduce the input data [129]. In our approach, the scoring function computes: (1) the excluded volume to avoid clashes between distinct residues/domains, (2) the quality of the covalent connectivity of the polypeptide chains, (3) the agreement with both the cryo-EM density map and with the CLMS data. Also, the scoring function takes into account the uncertainty of the input information, and encodes the presence of modularity and whether some data was transferred from another macromolecular sample. In our approach we prefer a Bayesian formulation of the scoring function [130], which is more objective than traditional scoring functions. This formulation can infer unknown quantities, combine different types of information and account for noise in the data. This is particularly important for the treatment of CLMS data, were some assignments might be erroneous.

The Bayesian scoring function is proportional to the posterior probability p(M|D,I) of a model M given the gathered data D and prior information I. The model M ≡ (X,{αi}) includes the structural coordinates *X* and additional parameters {*α_i_*} which describe unknown attributes of the data such as the noise or the relative weight of distinct data pieces. Using Bayes' theorem, the posterior probability is p(M|D,I) ∝ p(D|M,I)p(M,I), where the likelihood function p(D|M,I) is the probability of observing data *D*, given *I* and *M*, and the prior is the probability of model *M*, given *I*. The likelihood function for the CLMS data was built from a “forward model” that predicts the formation of a cross-link given the coordinates of a structural model, and a “noise model” that quantifies how much we can tolerate an error of the model (e.g. a cross-link that was observed and reported in the dataset which is not predicted to be formed in the model) [17], [24], [101]. Finally, the joint likelihood function p(D|M,I) for a dataset *D* = {*d_n_*} of *N_XL_* independently observed cross-links is the product of likelihood functions for each cross-link. Both the functions of the forward and the noise model introduce parameters to describe uncertainty. In particular, the likelihood function uses *ψ*, which is the unknown uncertainty that a cross-link is correctly assigned and regulates the weight of the corresponding restraint.

Importantly, the CLMS data was collected on a sample of Med-RNAPII Holoenzyme and transferred to the Mediator system lacking RNAPII. The addition of RNA pol II is observed to induce motions at the junctions between Mediator modules, while the intra-module topology appears unchanged [48], [131]. As a consequence, we expected the inter-module cross-links to be less accurate than the intra-module cross-links in describing the apo-Mediator state. To account for transferability and modularity, we assigned the inter- and intra-module cross-links, respectively 8% and 92% of the total, to different classes that were fit with different values of *ψ* (Fig. 4C). In this way we lessen the impact of inter-module cross-links while benefitting from intra-module ones.

Finally, the scoring function for the cryo-EM data was computed based on the Gaussian Mixture Model (GMM) representation of each domain, using the cross-correlation coefficient between GMM representations of the EM volume and model components [17]. The weights of each GMM component were normalized to the relative mass of the component vs the mass of the module. We exploited the modularity by dividing the EM density map into segments reflecting the Head, Middle and Tail module on the basis of previous approximate localization of subunits within two distinct regions of the EM map [70], [71] (Fig. 4B). During modeling, each Middle or Tail subunit density was restrained to the corresponding Middle or Tail EM density segments. The Head module was kept fixed in its initial docked position, except for the sections that were not resolved in the crystal structure and therefore present as coarse-grained representations. Recently, a Bayesian scoring function based on the GMM approach was developed, allowing an objective weighting of the EM density data with respect to the other information [128].

### 4.6 Exhaustive configurational sampling with replica exchange

One important requirement for the ISD approach is the generation of an exhaustive sampling of the model. To achieve this goal, we have to adopt improved sampling schemes and carry out a large number of independent sampling runs. In each run the positions and orientations of rigid bodies and flexible strings of beads, and the values of free Bayesian parameters, are randomly and iteratively perturbed in an effort to satisfy and optimize the scoring function, based on the Metropolis Monte Carlo algorithm. The improved sampling protocol followed the Replica Exchange scheme [130], using 64 replicas, with temperatures ranging between 1.0 and 2.5. The Replica Exchange scheme enhances the sampling by allowing hopping between alternative minima of the scoring function, thus preventing the sampling from becoming stuck in local minima. To confirm that we had sampled conformational space sufficiently to reach model convergence, we compared two independent halves of the solutions to each other and to the entire set, and assessed whether they display similar structural features. If we couldn’t find a satisfactory agreement, we double the number of sampling runs, until convergence. For the Mediator modeling, the exhaustive sampling produced a total of 165,523 models in 20 independent runs. In a recent paper, an automated convergence protocol was proposed with a series of statistical tests [132], which are more objective and stringent.

### 4.7 Model assessment and clustering in ISD

Integrative structural determination eventually results in clusters of individual models that best satisfy the input constraints. To identify these clusters, analysis is carried out on a small fraction of the models representing the very best scoring solutions. First, the 500 best scoring models (the solutions) are selected from the whole sampled ensemble and checked for whether they satisfy the input restraints. Second, the solutions are clustered by structural similarity using the root mean squared distance (RMSD) of the beads as a structural metric. The number of clusters is set to be the most parsimonious number that separates the main structural differences between all the solutions. Finally, the representative cluster(s) are selected according to their score, or alternatively the agreement with data that was not used in the scoring function (the validation data).

In the case of the Mediator, the solutions were grouped into four main clusters, with distinct structural properties. Clusters 1, 2, and 4 had the Middle module oriented in the expected orientation (i.e., Med1 and Med19 were at the bottom and at the top of the module, respectively). The three clusters differ in the arrangement of the Tail subunits. Cluster 3 had the Middle module in a ‘flipped’ orientation (i.e., Med1 and Med19 are at the top and at the bottom of the module, respectively). All the solutions from all clusters satisfied excluded volume, sequence connectivity, and EM restraints. Since the CLMS dataset was transferred from the Holoenzyme sample, not all the cross-links were satisfied. In fact, while 95% of intra-module cross-links were fulfilled, only a small portion (10%) of inter-modular cross-links were satisfied. The Bayesian scoring function automatically down-weighted the inter-modular cross-links to satisfy the EM restraint, pointing to an inconsistency of the inter-modular cross-links in the Holoenzyme dataset with the EM density map of the free Mediator. The satisfaction of intra-modular cross-links suggests that the arrangement of Mediator subunits within each module of the Holoenzyme retains the main characteristics to that in the free Mediator. Since the average scores of the different clusters were only marginally different, it was not possible to rank the clusters and identify the configuration which best fit the input data. Therefore, we used the validation data, and in particular the EM localization experiments, to choose the representative cluster. Remarkably, only Cluster 1, which also happened to be the top-scoring cluster, was fully in agreement with all EM localization experiments.

### 4.8 Calculating precision of the solution, subunit density and subunit-subunit proximities in ISD

The degree to which a component of the model, either a coarse-grained bead or residue, fluctuates across the ensemble of individual models indicates how precisely localized that component is in the modeling solution. We used several analysis metrics to represent the global and relative localization of domains and compute their precision: the localization density, the domain precision calculation, the root mean squared fluctuations (RMSF), and protein-protein contact analysis. First, for a given cluster of solutions, we computed the probability of finding any residue of a given protein at any point in space (i.e., the localization density map). The localization density is stored in a 3D grid, similarly to a 3D density map obtained from cryo-EM data, and it is a very convenient way to display the architecture of the whole complex from an ensemble of solutions. All localization density maps of proteins and domains are displayed in the software Chimera by an isosurface [133], which in our case was obtained using a threshold of 0.15. The precision of a domain (or a protein, or the whole complex) in a given cluster of solutions is calculated as the average RMSD between the cluster center and all other solutions. The precision can be used to compute the distance between two clusters, as the average RMSD on the whole complex, between every pair of solutions taken from the two clusters. The RMSF of a given residue is the standard deviation of the set of distances between the position of the residue in each solution of the cluster and its position in the cluster center.

As might be expected, there is a strong relationship between the number of consistent cross-links restraining a given bead and its localization precision. For instance, numerous cross-links were detected at the N-termini of Mediator Tail module subunits Med2 and Med3, with almost none at the C-termini. This was reflected in a RMSF near 10 Å for the N-terminal regions along with a high level of confidence in placing them at the junction between the Middle and Tail modules of Mediator (Fig. 3A). In contrast, after the first 100 residues or so it becomes difficult to localize these subunits. This intuitive finding points to the need to maximize the coverage of cross-links across the complex in question, particularly in regions with little or no atomistic structure available.

Protein-protein contact analysis of the solutions in a cluster can be carried out at the residue or domain level. In the former, the relative contact frequency of a pair of residues is computed based upon how often the two residues contact each other in the cluster, where a contact occurs when the distance between the residues is less than 10 Å. In the latter, two domains are in contact when the surface of any bead in one domain is within 10 Å of the surface of any bead in the second domain. Long sequences are divided into domains of 200 residues. Finally, the residue and domain interaction frequency can be rendered in a heat map that displays in a compact manner the propensity of contacts for a given cluster of solutions. Some of these contacts result simply from the identification of cross-links between the domains, however other contacts emerge indirectly due to the identification of synergistic sets of cross-links.

### 4.9 Model validation

The final and crucial stage of the whole ISD approach involves validating the models. Without validation, it is difficult to trust the models, as they can arise from artefacts of the modeling procedure itself, such as overfitting. First, the ensemble of solutions of the selected representative cluster is assessed in terms of how well it satisfies the data from which it was computed, including the cross-links, excluded volume, sequence connectivity and the three-dimensional EM restraints. This allows us to check the reliability of the modeling procedure: if models partially satisfy the data, one might suspect under-sampling, strong data inconsistency, or mistakes in the data-encoding in terms of the scoring function [14]. As mentioned above, the Mediator model fit the EM map, satisfied most of the intra-modular cross-link restraints, and it was stereochemically sound since it satisfied the excluded volume and sequence connectivity. Second, we used a cross-validation assessment using the CLMS dataset. In the cross-validation approach, we assessed the ensemble of solutions by comparing it with the ensemble of solutions obtained by jackknifing 10% the CLMS dataset in 34 different random ways. We compared the obtained localization maps, and computed the model precision to estimate the structural differences between clusters obtained using the whole CLMS dataset and clusters obtained using jackknifed datasets. Strikingly, the results were similar: both modeling approaches resulted in the same number of clusters, with the same structural features. That result suggested that the CLMS data was robust and accurate, and that the models were not a mere result of overfitting. Third, we validated the models based on unused data published in the literature. In fact, the Mediator model was remarkably consistent with almost all data from previous subunit interaction and subunit localization studies. The models explained subunit interactions inferred from co-expression [54], [67], [69], pulldowns/immunoprecipitation [54], [68], and yeast two-hybrid assays [49], [64], [65] with only three discrepancies, which were clearly spurious. Furthermore, the Mediator model was validated by comparison with results from two-dimensional EM studies that used subunit labelling or subunit deletions to map subunit locations. [70], [71]. The model was consistent with all these pieces of information, confirming the quality and the predictive power of the structure.

## 5 Conclusions and future perspectives on integrative structural techniques in the age of the Cryo-EM ‘resolution revolution’

Despite the success of cross-linking and low-resolution EM-derived integrative models at inferring both global architectures and pseudo-residue level structures of protein machines in recent years, there is a seemingly diminishing role for these workflows in the current era where high-resolution EM reconstructions of large protein complexes are becoming increasingly prevalent. What is the value of an imprecise model if we can seemingly wait another half year before an atomistic structure is revealed? Is there room to improve the performance of cross-linking based models? Since the precision of these models depends upon both the distance defined by the cross-linking reagent as well as the density of cross-links that constrain a given amino acid residue three-dimensionally, it should be possible to achieve more refined integrative models using “zero-length”, carbodiimide based cross-linkers [134] as well as by increasing the cross-linkable space with acid-directed [114] or other cross-linking chemistries. One approach to the latter aim is through using carbene generating, photochemical reagents [135]. However, the extremely heterogenous reaction mixtures that result from diazirine cross-linking make site-specific assignments of photo-cross-linked residues by mass spectrometry technically challenging and limit this approach. Recent attempts to utilize photo-cross-link derived restraints to aid protein structural prediction in the CASP11 competition showed no benefit [136]. However, technical developments using zero-length cross-linkers may still provide a future avenue to increasing the precision of cross-linking based structural inference. Such improvements may well be important for the structural analysis of protein complexes that continue to resist high-resolution EM reconstruction and thereby ensure that integrative modeling still has a role to play in the near future.

What factors determine the resistance of a protein complex to high resolution EM reconstruction? Even though cryo-EM has advanced significantly, heterogeneity derived from non-discrete, continual motions of domains still provides a significant challenge to algorithms designed to classify particles in 2- and 3-dimensions. This is especially the case when more than one region of a large multi-subunit complex displays continuous motion and the mobile regions represent a large proportion of the mass of the complex [137]. Many biological systems will be difficult to solve at atomic resolution for this reason and so the combination of low-resolution EM with X-ray crystallography and cross-linking will still play an important role in these circumstances. Some features of the Mediator complex that were first revealed through our integrative model such as the connection between the N-terminus of Med17 and the Middle module were later shown to be highly accurate by high-resolution EM [75] and crystallography [76]. For other features, our model remains the best description of Mediator available. These include the Tail module which is highly dynamic, contains many disordered regions, and participates in ”fuzzy” interactions with activators such as Gcn4 (see Section 3.6). Thus, a high-resolution EM reconstruction of the Tail module might not be possible with existing technology, but ISD approaches can infer molecular features at pseudo-residue level precision. For instance, our Tail module model predicts specific structural features such as the N-terminal portion of Med5 extending across the β-propeller domain of Med16 to contact Med15 [17] and associates a precision (RMSF value) with these predictions. Much of the Tail module remains resolved at poor resolution in our model due to lack of adequate cross-linking restraints. Hence, our structural understanding of the Tail module would likely be improved by the application of cross-linking methodologies that increased coverage in these domains.

In addition to the high frequency fluctuations of the Tail module, our data suggest that it also undergoes a more coordinated rearrangement upon PIC binding that places Med5 in contact with Med1 of the Middle module (Section 3.6). This represents a more stable conformational heterogeneity that is resolvable by EM. CLMS can aid in understanding these states through the use of quantitative MS methods. For instance, the Med-PIC can be cross-linked in the presence and absence of Gcn4 using reagents with different isotopic signatures [138]. The cross-linked samples are combined, prepared for mass spectrometry analysis, where the isotopic ratio of cross-linked peptide precursors reports on states that are enriched or depleted in the presence of Gcn4. Quantitative CLMS (qCLMS) can be extended to multiple states along a mechanistic pathway, such as promoter escape, through using multiplexed proteomics methods such as PRM [139] or TMT labelling [140]. Using such methods, sub-conformations of a protein assembly do not need to be homogenous, as long as one state is enriched sufficiently to be quantified by MS. Hence, these sorts of qCLMS approaches as inputs to ISD might be useful in resolving more dynamic and heterogenous populations of protein assemblies as well, although they do require that the individual mechanistic states can be partially stabilized through modulating the sample conditions, without significant changes to the protein composition.

However, the true value of integrative models going forward lies not in traditional structural biology, which focuses on elucidating stable elements of macromolecular tertiary and quaternary structure, but as tools to study the non-classical, sequence-independent and low-affinity interactions known as “fuzzy-interactions” [141], [142] and protein quinary structure [143], [144], [145]. These emerging areas of study have been implicated in transcriptional regulation through the binding of gene specific activators to their co-activator targets in complexes without a single ground-state conformation, and by governing the formation and organization of cellular structures that sequester large ensembles of transcriptional machinery in sub-nuclear, membrane-less compartments. These processes are inherently conformationally dynamic and heterogenous and thus not easily represented by single-state atomistic molecular models that can be resolved by high-resolution EM or X-ray derived techniques. Instead, we anticipate that multi-state representations of these systems will be accessible via extension of existing integrative modeling methodology. Experimentally, techniques that are well integrated into integrative methods such as CLMS and mixed resolution EM will continue to play an important role, as will other methods such as NMR [146], [147], real-time immunofluorescence microscopy [148], proximity labelling-MS [149], and chemical footprinting-MS methods [150] such as hydrogen-deuterium exchange or oxidative footprinting.

Quinary structure represents a fifth level of protein organization based on transient and weak-affinity protein interactions [143]. These fleeting interactions are difficult to capture experimentally, yet they play a major role in organizing the intracellular environment at a scale larger than a protein complex yet smaller than an organelle. For instance, various multi-step metabolic pathways rely on the spatial co-localization of multiple macromolecular complexes for efficient biosynthesis. Membrane-less compartments have been shown to form in the nucleus to sequester molecules with related functions [151]. Examples include: Nuclear speckles, which contain mRNA splicing factors, the Nucleolus, which contains ribosome synthesis machinery, and super-enhancers, which bring together extremely high concentrations of transcriptional machines including activators, co-activators and the PIC, at promoters that seem to have particularly important roles in regulating cellular differentiation and oncogenesis [40]. In lieu of phospholipid membranes, these microenvironments are defined by the oligomerization of multi-valent scaffold proteins that show a high prevalence of intrinsically disordered domains with low sequence complexity [152]. For instance, heterochromatin is dynamically sequestered by the multivalent heterochromatin protein HP1α, which contains two structurally disordered regions. Phosphorylation at one of these domains regulates extension at the other to a conformation that favours inter-HP1α interactions that act as a scaffold to condense and isolate transcriptionally silent regions of the genome [153]. Membrane-less compartments exhibit properties of liquid droplets, such as phase separation and droplet fission and fusion [143]. While the free energy of these interactions is weak (1 kcal/mol, an order of magnitude lower than quaternary protein structure), the densely crowded cellular environment (200–400 g/L protein concentration) amplifies the chemical forces exerted by neighbouring molecules through electrostatic, hydrophobic, polar, and hydrogen bonding interactions. Hence, small chemical modifications can lead to large-scale re-organization of the cellular microenvironment.

In addition to contributing to the characterization of membrane-less compartments, ISD will also likely play an important role in understanding transcription complexes at higher levels of organization. In addition to the Mediator-PIC assembly, numerous protein complexes act at a gene promoter to influence its transcription. These include other co-activators such as TFIID and SAGA as well as chromatin remodeling complexes such as SWI/SNF and RSC, and inhibitory modules such as the Mediator Kinase module. How these complexes coordinate their actions with the Med-PIC, the nature of their super-assemblies if any, and how promoter identity influences mechanism are open questions. These larger assemblies represent a logical next step in continuing our modular approach to ISD which builds models of increasingly larger assemblies based in part on transferability of data acquired on smaller subassemblies. However, unlike previous iterations of this process, these larger assemblies are governed by quinary interactions. Super enhancers are clusters of spatially proximate enhancers that regulate a single promoter and contain elevated concentrations of Mediator, the general transcriptional machinery and other co-activators relative to regular promoters [40]. Various groups have proposed that super enhancers represent phase separated droplets which sequester high concentrations of transcriptional machinery at gene promoters of particular importance to oncogenesis and determination of cellular fate [41], [42], [154]. Mediator is enriched in super enhancers and antibodies directed at Middle module subunit Med1, which contains a high portion of low complexity domains, are used to characterize super enhancers in ChIP-Seq or fluorescence imaging experiments. The organization and composition of super-enhancers is very much unknown. For instance, what is the nature of the scaffold that creates a phase-separated compartment and what are the client proteins? The stoichiometry of these compartments is also unknown and likely to vary, and it is likely that organization of the individual protein machines within the compartment is governed by weak interactions with multiple modes of binding. Hence, it is unlikely that EM will ever be able to produce a single, atomistic ground state structure of a super-enhancer. However, ISD approaches which describe biological systems probabilistically and can address multi-state systems, can potentially be developed to model super enhancers and other conglomerations of transcription complexes in a way that yield clues as to the structural principles that influence gene regulation. Since these complexes are good examples of quinary structures that will be very labile in nature, integrative structural approaches will likely play a key role in advancing the structural biology of these complexes, as it did in the emerging picture of the Mediator complex and related transcription complexes.

The value of integrative models in the future is in addressing problems in structural biology that are not tractable by high-resolution methods. These are problems involving heterogenous structures, highly dynamic conformational fluctuations, intrinsically disordered domains, “fuzzy”-interactions, and higher-order structures within a cell encompassing ensembles of transcription related protein complexes. These areas comprise many emerging themes in transcriptional regulation, and they challenge existing structural biology techniques and paradigms which are best suited for stable, homogenous assemblies.
